# PKM2 is not required for colon cancer initiated by APC loss

**DOI:** 10.1186/s40170-017-0172-1

**Published:** 2017-11-30

**Authors:** Allison N. Lau, William J. Israelsen, Jatin Roper, Mark J. Sinnamon, Larissa Georgeon, Talya L. Dayton, Alissandra L. Hillis, Omer H. Yilmaz, Dolores Di Vizio, Kenneth E. Hung, Matthew G. Vander Heiden

**Affiliations:** 1 Koch Institute for Integrative Cancer Research and the Department of Biology at Massachusetts Institute of Technology, Cambridge, MA 02139 USA; 2 Department of Biochemistry, University of Texas Southwestern Medical Center, Dallas, TX 75390 USA; 3 Department of Medicine, Tufts Medical Center, Boston, MA 02111 USA; 4 Center for Systems Biology, Massachusetts General Hospital, Boston, MA 02114 USA; 5 Harvard Medical School, Boston, MA 02114 USA; 6 Department of Pathology, Massachusetts General Hospital, Boston, MA 02114 USA; 7 Departments of Surgery, Biomedical Sciences, and Pathology and Laboratory Medicine, Cedars-Sinai Medical Center, Los Angeles, CA USA; 8 Department of Medical Oncology, Dana-Farber Cancer Institute, Boston, MA 02115 USA

**Keywords:** PKM2, APC, β-catenin, Colon cancer

## Abstract

**Background:**

Cancer cells express the M2 isoform of the glycolytic enzyme pyruvate kinase (PKM2). PKM2 expression is not required for some cancers, and PKM2 loss can promote cancer progression; however, PKM2 has been reported to be essential in other tumor contexts, including a proposed non-metabolic role in β-catenin nuclear translocation. PKM2 is expressed in colon cancers where loss of the *Apc* tumor suppressor results in β-catenin nuclear translocation and aberrant activation of the canonical Wnt signaling pathway. Whether PKM2 is required in this colon cancer context has not been investigated.

**Results:**

Colon tumorigenesis was induced in mice harboring conditional *Apc* and *Pkm2* alleles, and tumor progression was monitored by serial colonoscopy. PKM2 deletion had no effect on overall survival, the number of mice that developed tumors, or the number of tumors that developed per animal. Immunohistochemical analysis demonstrated PKM2 expression in wild-type tumors and the expected loss of PKM2 expression in tumors from *Pkm2* conditional mice. Loss of PKM2 resulted in pyruvate kinase M1 expression but had no effect on nuclear β-catenin staining. These findings are consistent with tumor growth and activated Wnt signaling despite PKM2 loss in this model. We also found a large fraction of human colon cancers had very low or undetectable levels of PKM2 expression.

**Conclusions:**

PKM2 is not required for *Apc*-deficient colon cancer or for nuclear translocation of β-catenin in *Apc*-null tumor cells. These findings suggest that PKM2 expression is not required for colon tumor formation or progression.

**Electronic supplementary material:**

The online version of this article (10.1186/s40170-017-0172-1) contains supplementary material, which is available to authorized users.

## Background

Pyruvate kinase is an enzyme that catalyzes the last reaction of glycolysis to produce pyruvate and ATP from phosphoenolpyruvate and ADP. The enzymatic activity of the M2 isoform of pyruvate kinase (PKM2) is regulated by growth signaling and multiple allosteric effectors, coupling PKM2 activity to the metabolic and signaling state of the cell in order to promote proliferative metabolism in appropriate contexts [1, 2]. PKM2 is expressed in a wide variety of cancer types and both metabolic and non-metabolic functions for PKM2 in cancer have been suggested [3]; however, the requirement for PKM2 in different cancer contexts remains controversial [4]. For instance, PKM2 has been reported to play a role in driving cancer cell proliferation via signaling functions that are distinct from a role in glycolysis [5–7], including a nuclear function to activate β-catenin downstream of growth signaling [8]. However, other work has shown that PKM2 is dispensable for the growth and maintenance of xenograft tumors [9], that loss of PKM2 can promote progression of BRCA1 loss-driven breast cancer [10] and medulloblastoma tumor growth [11], and that PKM2 is not required for leukemia or liver tumor formation [12, 13].

PKM2 is expressed in colon cancers [14] and has been reported to facilitate colon cancer cell proliferation, migration, and the epithelial-mesenchymal transition [15–17]. Immunohistochemical analysis of human colon tumors revealed that tumors express higher levels of PKM2 as compared to paired normal colon tissue and that increased PKM2 expression is associated with greater tumor stage and with lymph node metastasis [15]. Due to the controversy surrounding the requirement for PKM2 by tumors and the functions of this enzyme in cancer, we tested the importance of PKM2 in colon cancer by crossing mice harboring a conditional *Pkm2* allele [10] to mice with conditional alleles of the *Apc* (*Apc* ^*CKO*^) tumor suppressor gene [18]. This genetically engineered mouse model of colon cancer has been applied to study the role of oncogenic *Kras* and *Braf* in tumorigenesis [19–22]. By comparing mice with and without *Pkm2* conditional alleles in this model, we sought to determine whether PKM2 is essential for colon cancer initiation and progression following *Apc* loss.

## Results

**Fig. 1:**
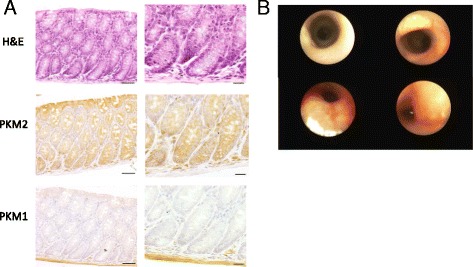
PKM2 is expressed in colon epithelial cells where loss of APC results in tumor formation. **a** FFPE colon tissue sections from mice were stained with hematoxylin and eosin (H&E) and isoform-specific antibodies against PKM2 or PKM1 as shown. Scale bars represent 50 μm for all images at ×20 magnification (left) and 20 μm for all images at ×40 magnification (right). **b** Adenoviral Cre was used to induce colon tumors, with ligation-restricting adenoviral Cre exposure to the distal colon. Lesion appearance and tumor progression were monitored by serial colonoscopy as shown. Representative tumors from four different *Apc* ^flox/flox^ *Pkm2* ^flox/flox^ mice are shown at various stages of progression

PKM2 is expressed in many normal tissues, including the intestine [23], while the pyruvate kinase M1 (PKM1) isoform, encoded by an alternative splice product of the PKM gene, is also expressed in the intestine [14]. To determine which cell types of the colon express each isoform, we performed immunohistochemistry on normal mouse colon tissue using PKM2 and PKM1 isoform-specific antibodies [10]. PKM2 staining was observed in the epithelial cells of the crypts, including crypt base cells that include colon stem cells, but was not prominent in the lamina propria (Fig. 1a). The muscularis propria layer expressed PKM1 but not PKM2 (Fig. 1a). This makes it likely that tumors in this model arise from PKM2-expressing cells.

To generate *Apc*-deficient colon tumors, colon tissue from *Apc* ^*CKO*^ mice with and without homozygous conditional *Pkm2* alleles was exposed to adenoviral Cre, and tumor growth, latency, and multiplicity per animal were determined. In this method, a section of distal colon is transiently ligated and adenovirus-encoding Cre recombinase is introduced into the lumen of the colon. Cre expression in infected cells results in Apc loss and stochastic tumor initiation in the distal colon that can be monitored with optical colonoscopy [18]. Representative colonoscopy images showing colon tumors at various stages of progression are shown in Fig. 1b.

**Fig. 2:**
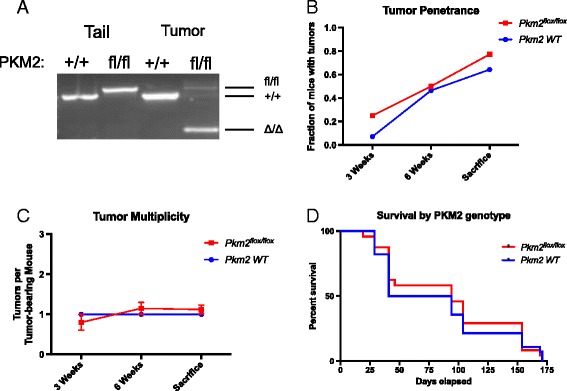
PKM2 deletion does not affect tumor penetrance, multiplicity, or survival in mice with APC deletion. **a** Colon tumors were initiated in *Apc* ^flox/flox^ *Pkm2* ^+/+^ and *Apc* ^flox/flox^ *Pkm2* ^flox/flox^ mice, and PCR genotyping of the Pkm2 allele in tumors arising in each condition is shown. Analysis of tail DNA from *Pkm2* ^*+/+*^ and *Pkm2* ^*flox/flox*^ mice is shown as a control. **b** Tumor penetrance in *Apc* ^flox/flox^ *Pkm2* ^+/+^ and *Apc* ^flox/flox^ *Pkm2* ^flox/flox^ mice was determined by colonoscopy. The fraction of mice with tumors did not differ significantly between cohorts at 3 weeks post-infection, at 6 weeks post-infection, or at final inspection (*n* = 24 *Pkm2* ^flox/flox^ mice, 28 *Pkm2* ^+/+^ mice per cohort, *p* > 0.1 at all time points, Fisher’s exact test). **c** *Apc* ^flox/flox^ *Pkm2* ^flox/flox^ mice had similar tumor multiplicity when compared to *Apc* ^flox/flox^ *Pkm2* ^+/+^ mice. Multiplicity is defined as the number of tumors per tumor-bearing animal (*n* = 17 *Pkm2* ^flox/flox^ mice, 18 *Pkm2* ^+/+^ mice, *p* > 0.25). **d** Kaplan-Meier curve showing survival of the *Apc* ^flox/flox^ *Pkm2* ^+/+^ and *Apc* ^flox/flox^ *Pkm2* ^flox/flox^ cohorts. Pkm2 deletion had no statistically significant effect on survival (*n* = 24 *Pkm2* ^flox/flox^ mice, 28 *Pkm2* ^+/+^ mice per cohort, *p* = 0.7060, Log-rank (Mantel-Cox) test)

To verify that *Pkm2* was deleted in tumors from *Apc* ^*flox/flox*^;*Pkm2* ^*flox/flox*^ mice, we analyzed genomic DNA from *Apc* ^*flox/flox*^;*Pkm2* ^*+/+*^ and *Apc* ^*flox/flox*^;*Pkm2* ^*flox/flox*^ colon tumors to confirm that PKM2 recombination had occurred (Fig. 2a). There was no significant difference in the fraction of mice developing one or more tumors 3 weeks, 6 weeks, or at later time points following Cre administration in *Apc* ^*flox/flox*^;*Pkm2* ^*flox/flox*^ mice compared with control *Apc* ^*flox/flox*^;*Pkm2* ^*+/+*^ mice (Fig. 2b). There were also no differences in the number of tumors arising per mouse (Fig. 2c) over the same period of time. Overall survival was not significantly different between *Apc* ^*flox/flox*^;*Pkm2* ^*+/+*^ and *Apc* ^*flox/flox*^;*Pkm2* ^*flox/flox*^ mice with *Apc* loss driven colon tumors (Fig. 2d).

**Fig. 3:**
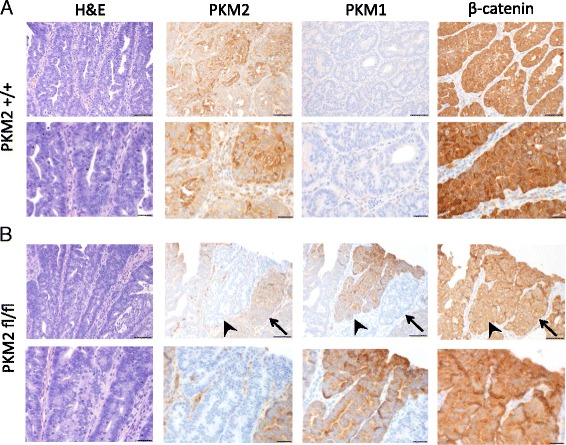
PKM2 expression is lost in colon tumor cells in *Pkm2* conditional animals. **a** Tissue sections from tumors arising in *Apc* ^flox/flox^ *Pkm2* ^+/+^ mice were stained with hematoxylin and eosin (H&E) or with antibodies against PKM2, PKM1, or β-catenin as shown. **b** Colon tumors arising in *Apc* ^flox/flox^ *Pkm2* ^flox/flox^ mice were stained with hematoxylin and eosin (H&E) or with antibodies against PKM2, PKM1, or β-catenin as shown. A PKM2 negative region that switches to PKM1 expression is marked with an arrowhead, and a PKM2 positive region is marked with an arrow. Scale bars represent 50 μm for all images at ×20 magnification (top row in each panel) and 20 μm for all images at ×40 magnification (bottom row in each panel)

We also analyzed pyruvate kinase expression in tumors isolated from these mice using immunohistochemistry with pyruvate kinase isoform-specific antibodies. As expected, analysis of colon lesions showed that PKM2 wild-type tumors expressed almost exclusively PKM2 (Fig. 3a). Similar to breast tumors [10], some of these PKM2 wild-type tumor cells appeared to express very little PKM2 despite a lack of PKM1 expression (Fig. 3a, Additional file 1: Figure S1A). PKM2 expression in normal colon tissue and PKM1 expression in the lamina propria were confirmed in these same tissue sections (Additional file 1: Figure S1B). Colon tumors from *Apc* ^*flox/flox*^;*Pkm2* ^*flox/flox*^ mice showed loss of PKM2 expression in areas of the tumors; however, PKM2 expression was maintained in some areas and in normal colon tissue (Fig. 3b). Examination of serially stained sections showed that tumor areas with loss of PKM2 displayed expression of PKM1 (Fig. 3b), findings similar to what was previously observed in both tumor and normal tissue [10, 12, 13].

The APC tumor suppressor protein is a negative regulator of canonical Wnt signaling. APC loss results in nuclear translocation of β-catenin, and strong nuclear β-catenin staining is observed in this colon cancer model [18]. Because work in glioblastoma has suggested that PKM2 is required for β-catenin activation downstream of EGF stimulation in that tissue [8], we determined whether loss of PKM2 impacted β-catenin localization in tumors arising in *Apc* ^*flox/flox*^;*Pkm2* ^*flox/flox*^ mice (Fig. 3a, b, Additional file 1: Figure S1C). β-catenin staining was found only at the plasma membrane of non-transformed colon epithelial cells (Additional file 1: Figure S1C). In contrast, both PKM2 wild-type and PKM2-null tumor cells showed strong nuclear β-catenin staining (Fig. 3a, b, Additional file 1: Figure S1C). These data suggest that PKM2 is not required for nuclear translocation of β-catenin downstream of APC loss in colon cancer.

**Fig. 4:**
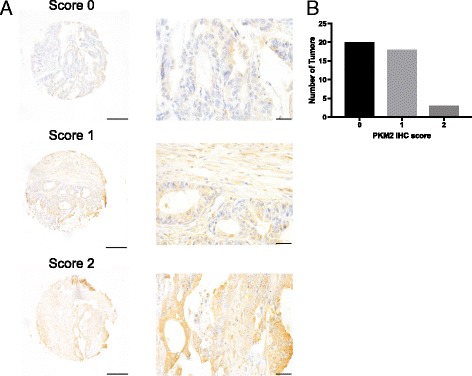
Human colon tumors express low levels of PKM2. **a** Representative TMA cores containing human colon tumors showing PKM2 staining scored as 0, 1, or 2. Scale bars represent 50 μm for all images at ×20 magnification (left) and 20 μm for all images at ×40 magnification (right). **b** Distribution of PKM2 staining intensities for 41 human colon tumors

To examine PKM2 expression in human colon tumors, we performed PKM2 immunohistochemistry on 41 colon tumor tissue array samples. Nearly half of the samples (20/41) were negative for PKM2 expression, and 18/41 expressed low levels of PKM2 (Fig. 4a, b). The lack of expression of PKM2 in human colon tumors further supports a model in which PKM2 upregulation is not required and is instead dispensable for the formation and growth of at least some colon tumors.

## Discussion

Taken together, these results argue that PKM2 is not required for APC-deficient colon cancer. These findings are consistent with previous results suggesting that PKM2 is not required for BRCA1-deficient breast cancer [10], medulloblastoma [11], BCR-ABL-driven leukemia [12], or hepatocellular carcinomas with diverse genetics that arise in animals with metabolic syndrome [13]. While loss of PKM2 slowed leukemia progression, deletion of PKM2 increased breast cancer and medulloblastoma progression and could promote hepatocellular carcinoma. We find here that the absence of PKM2 does not prevent sporadic colon tumor initiation and progression, nor does it affect tumor multiplicity or survival. PKM2 expression was low and variable in PKM2 wild-type tumors, as well as in human colon tumors. An isoform switch from PKM2 to PKM1 expression was observed in PKM2-null tumor regions, findings that are reminiscent of those observed in BRCA1 loss-driven breast cancer [10] or leukemia [12] models, and argue that dispensability of PKM2 is not limited to specific tumor types or to individual driver mutations. Recent work has shown that PKM2 is also dispensable during normal embryonic development, further supporting the idea that PKM2 is not necessary for cell proliferation in various physiological contexts [13].

Since PKM1 is constitutively active, whereas PKM2 activity can change between low activity and high-activity states, PKM1 expression has been associated with higher pyruvate kinase activity and decreased proliferation [10, 24]. Although we did not measure overall pyruvate kinase activity in PKM2 wild-type versus PKM2-null tumors, we did observe higher PKM1 expression in PKM2-null tumors. In contrast to other models where PKM2 has been deleted [24, 25], PKM1 expression following PKM2 deletion in the colon did not appear to affect tumor progression or proliferation. This may be related to the possibility that not all tumor cells efficiently deleted PKM2, as PKM2 expression was retained in some areas of the tumor, likely due to the multi-clonal nature of colon tumors in this model. The pattern of retained PKM2 expression is most consistent with inefficient deletion of PKM2 in some founder clones, as mixed cell populations of PKM2-expressing and deleted cells were not observed. This argues against a selective pressure to lose PKM2 expression in promoting tumor growth following APC loss. Nevertheless, the loss of PKM2 expression in some tumor areas suggests that PKM2 is not required for tumor formation in this model.

Our results demonstrate that nuclear localization of β-catenin downstream of canonical Wnt signaling is not dependent on PKM2. PKM2 has been reported to play a role in nuclear β-catenin localization through a Wnt-independent mechanism downstream of EGF receptor signaling in glioblastoma [8]. Therefore, it is possible that PKM2 could play a role in β-catenin nuclear localization driven by EGF signaling in colon cancers where this pathway is hyperactive. Altered EGF receptor signaling can be a driver of colon cancer [26], and whether PKM2 has a different requirement in this context is not known. Nevertheless, these data argue against the requirement for a unique PKM2 activity in malignancy outside of specific contexts [6, 7, 27, 28]. Instead, the findings support a model where PKM2 does not have a unique role but instead allows cancer cells to integrate metabolic and signaling inputs to support tumor growth.

## Conclusions

Our study found that PKM2 is not required for colon tumor initiation or growth driven by APC loss. Loss of PKM2 in APC-deficient tumors resulted in higher PKM1 expression but did not change β-catenin expression patterns, further arguing that PKM2 is not required for β-catenin nuclear localization downstream of APC loss. Many human colon tumors examined in this study also were found to have very low or undetectable PKM2 expression, also supporting the conclusion that PKM2 is dispensable for the growth of some colon tumors.

## Methods

### Mouse models and tumor induction

*Pkm2* ^flox/flox^ mice [10] were bred to the *Apc* ^*CKO*^ mice [18] to generate animals of relevant genotypes. Ad5CMVcre adenovirus was obtained from the Gene Transfer Vector Core, University of Iowa. Surgeries to deliver Ad5CMVcre adenovirus to the colon were performed as described previously [18].

### Colonoscopy

Mice were fasted overnight and anesthetized using 2% isoflurane. The colon was washed with PBS and colonoscopy was performed using a custom-made system as described previously [18].

### Immunohistochemistry

Sections from formalin-fixed paraffin-embedded tissue were stained with hematoxylin and eosin or with antibodies against PKM2 (Cell Signaling Technologies #4053, 1:800 dilution), PKM1 (Cell Signaling Technologies #7067, 1:1000 dilution), or β-catenin (BD Transduction Laboratories #610153, 1:100 dilution) as previously described [10, 18]. We analyzed a multi-tissue tissue microarray (TMA) containing tissue obtained from the archives of the Institute of Pathology at the University of Basel [29, 30]. The TMA was scored for IHC intensity independently by both a pathologist (D.D.V.) and another member of the team (T.L.D.). Tumors that showed no positive staining for PKM2 were given a score of 0, those with weak staining were given a score of 1, and tumors with strong PKM2 staining were given a score of 2.

## Additional file

Additional file 1: Figure S1. Additional immunohistochemistry images. A) PKM2 wild-type tumors show variability in PKM2 staining; representative images of PKM2 staining in tumors from *Apc* ^flox/flox^ *Pkm2* ^+/+^ mice. B) PKM2 staining of normal colon and PKM1 staining of muscle fiber from the same sections of *Apc* ^flox/flox^ *Pkm2* ^+/+^ mice shown in Fig. 3a. C) β-catenin staining in normal areas (left panel, right panel marked with an arrowhead) and tumor tissue (right panel, marked with an arrow) of *Apc* ^flox/flox^ *Pkm2* ^+/+^ mice. Scale bars represent 50 μm for all images at ×20 magnification (left for each panel) and 20 μm for all images at ×40 magnification (right for each panel). (DOCX 1650 kb)

## Abbreviations

PKM1: Pyruvate kinase, M1 isoform
PKM2: Pyruvate kinase, M2 isoform
