# Supplementary material for: PKM2 is not required for colon cancer initiated by APC loss
Source: Cancer Metab. 2017 Nov 30;5:10. doi: 10.1186/s40170-017-0172-1 (PMC5707917; doi:10.1186/s40170-017-0172-1)
Supplement: Supplementary file 1 [file 40170_2017_172_MOESM1_ESM.docx]

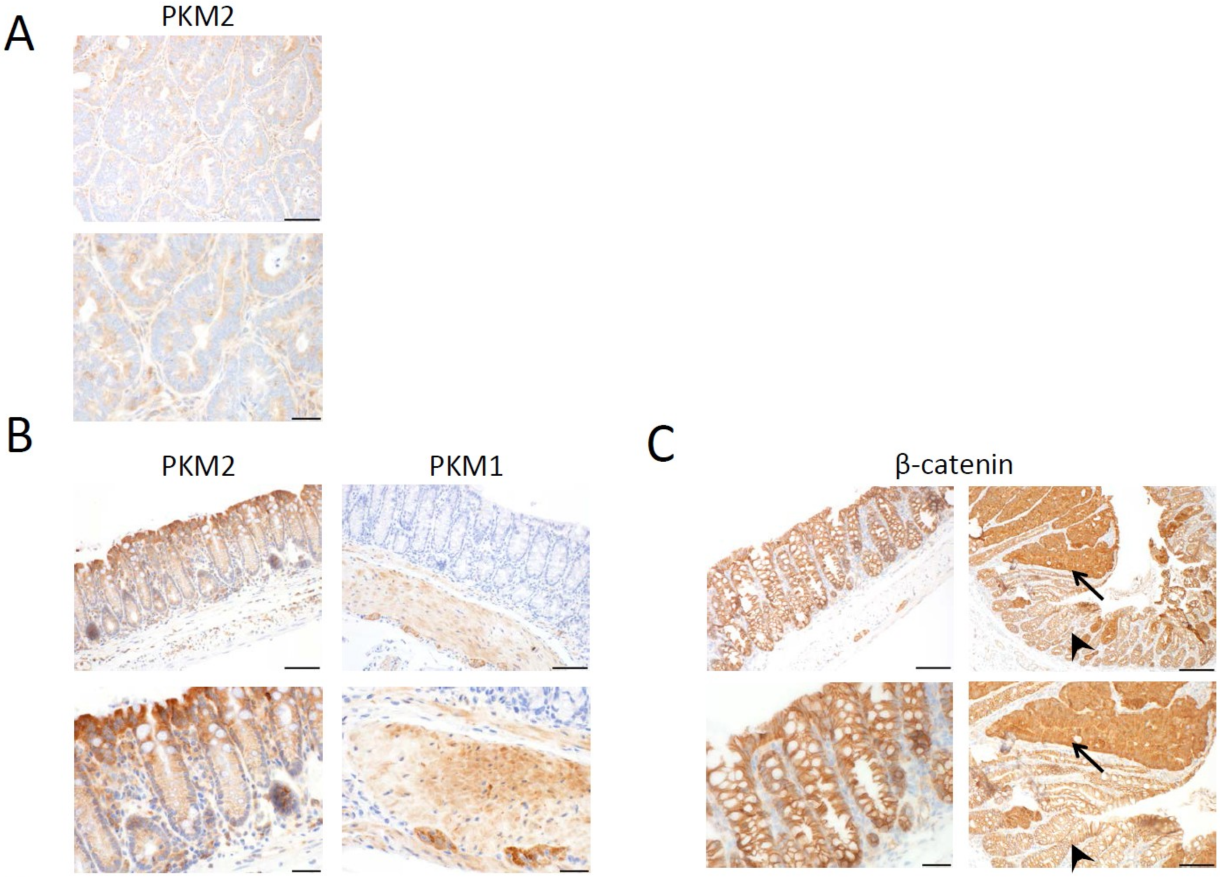

Figure S1
